# Role of Urine Dipstick Screening in the Diagnosis of Pediatric Urinary Tract Infection in an Urban Referral Centre in Tamil Nadu, India

**DOI:** 10.7759/cureus.107712

**Published:** 2026-04-25

**Authors:** Jane Allen Christa Arokiadas, Vasanthakumar Manokaran, Madhivanan Arulmozhi, Reshma I

**Affiliations:** 1 Department of Pediatrics, Vinayaka Mission's Medical College and Hospital, Vinayaka Mission’s Research Foundation Deemed to be University (DU), Karaikal, IND; 2 Department of Community Medicine, Sri Manakula Vinayagar Medical College and Hospital, Puducherry, IND; 3 Department of Pediatrics, NeoPrime Hospitals, Erode, IND

**Keywords:** children, diagnostic test, screening, urinary tract infection, urine dipstick test

## Abstract

Introduction: Urinary tract infection (UTI) is the most prevalent bacterial illness among children and a common cause of fever without an identifiable focus. Diagnosing UTI in children can be challenging, as their symptoms are nonspecific. Timely and accurate diagnosis is essential to prevent renal damage and reduce unnecessary interventions. Dipstick tests offered a rapid, non-invasive, and cost-effective alternative to urine culture, particularly in resource-limited settings.

Objectives: This study aimed to evaluate the diagnostic performance of urine dipstick screening, specifically nitrite and leukocyte esterase tests, in detecting pediatric UTIs in an urban referral centre in Tamil Nadu.

Methodology: A cross-sectional diagnostic study was conducted among 140 children aged two months to 12 years, presenting with symptoms suggestive of UTI. Urine samples were collected under aseptic conditions and tested using dipstick strips, microscopy, and culture. Nitrite and leukocyte esterase served as surrogate markers, enabling early identification and triage.

Results: Among the 140 children enrolled, infants constituted the largest age group (37.9%), and fever was the predominant symptom, reported in 90% of cases. The sensitivity and specificity of nitrite was 30% and 97.5%, whereas leucocyte esterase showed values of 55% sensitivity and 85% specificity. When compared to urine culture, the combined Nitrite and Leucocyte esterase test has good specificity (99.2%) and negative predictive value (89.5%) to rule out UTI, when both markers are negative.

Conclusion: Urine dipstick screening can effectively rule out UTI and help in the identification of children requiring further diagnostic evaluation, reducing unnecessary cultures and facilitating prompt treatment, especially in limited laboratory settings. By streamlining diagnosis and minimizing delays, dipstick screening contributes to improved pediatric UTI management and prevention of long-term complications.

## Introduction

Urinary tract infection (UTI) refers to colonization of a pathogen occurring at any site along the urinary tract, including the kidney, ureter, bladder, and urethra. It is recognized as the most common bacterial infection in children under two years of age and is a leading cause of serious bacterial infection in those with fever of unknown origin. The prevalence of urinary tract infection varies between 4.1% and 7.5%, making it the commonest bacterial illness among febrile infants and young children [1]. In older children less than 19 years, with urinary symptoms, the pooled prevalence of UTI, both febrile and afebrile, was 7.8% (CI: 6.6-8.9) [2]. The lifetime risk of developing a UTI before the age of 14 years is approximately 1-3% in boys and 3-10% in girls [3]. Further, the recurrence rates range from 12% to 30% in the first 6-12 months following an initial episode [4,5]. Key risk factors for recurrence include age less than six months at first diagnosis of UTI [6], children aged 2-6 years, especially those between 3-5 years, and vesicoureteral reflex grades 3-5 [7].

UTI has a variable symptomatology. The clinical diagnosis of UTI is often difficult due to nonspecific symptoms occurring in children. Hence, the diagnostic studies play an important role in accurately identifying the affected individuals. Over time, diagnostic approaches have evolved towards non-invasive, rapid methods for quicker and more accurate diagnosis. Accurate diagnosis is essential not only to identify, treat and evaluate the child at risk of renal damage, but it is also essential to avoid unnecessary treatment in those not at risk of renal damage. This will bring down the need for unnecessary interventions and treatment [8]. Even though the long-term follow-up data are limited, one study has reported the UTI complication among children with renal scarring from pyelonephritis, in which 23 % developed hypertension and 10 % developed end-stage renal disease [9]. Notably, younger children less than two years of age are at greater risk of parenchymal defects than older children [10].

Urine testing in diagnostic laboratories typically involves microscopy, Gram staining, automated assays, and urine cultures. Although culture remains the gold standard, it is expensive and time-consuming, requiring approximately 18 hours for organism growth and up to 72 hours for final results. In contrast, reagent strip tests provide a quick, non-invasive, and efficient alternative method for early detection of UTIs, particularly in emergency and community settings, where timely treatment initiation is critical. Dipstick tests detect surrogate markers such as leukocyte esterase and nitrite, offering a cost-effective, time-efficient option for smaller laboratories that lack culture facilities [11]. While suprapubic aspiration and transurethral catheterization are ideal for young children, they are often impractical in outpatient settings, emphasizing the need for rapid tests [12,13]. Nitrite and leukocyte esterase serve as indirect indicators of UTI [14] and are especially useful for screening in primary health centres and school health services, enabling faster diagnosis than conventional urine culture.

In addition, no trained staff or well-equipped laboratory is required for dipstick methods. Rapid urine dipsticks effectively identify cases that need urine culture, thereby reducing unnecessary diagnostic tests and expenditure. Cultures can then be reserved for children with high clinical suspicion of UTI and positive dipstick results.

While there are studies on this topic, very few have focused on the Indian pediatric population. Hence, this study aimed to investigate the role of the urine dipstick test in the diagnosis of urinary tract infection in children aged two months to 12 years attending an urban referral centre in Tamil Nadu.

The study was conducted with the following objectives: 1. To determine the diagnostic performance of urine dipstick tests (nitrite and leukocyte esterase), individually and in combination, in the diagnosis of UTI; 2. To compare urine dipstick findings with urine microscopy and culture; 3. To identify the clinical profile, risk factors, and causative organisms associated with UTI.

## Materials and methods

This cross-sectional diagnostic study was conducted in the Department of Pediatrics, CSI Kalyani Multi-Specialty Hospital, Mylapore, Chennai, for a period of one year from January 2020 to December 2020. Children aged two months to 12 years who attended the Pediatric OPD or were admitted to the ward were recruited to the study after obtaining the institutional ethics approval. Based on feasibility and the expected patient load, a total of 140 children presenting with symptoms suggestive of UTI were included in the study.

We recruited children consecutively into the study if they presented with any of the following symptoms like fever without an obvious focus for more than 48 hours (based on history or clinical examination or rectal temperature > 39^o^C), nonspecific symptoms such as failure to thrive, irritability, crying while passing urine, decreased urine output, vomiting and malodorous urine, or specific symptoms like abdominal pain, dysuria, haematuria, frequent micturition and urinary incontinence.

**Figure 1:**
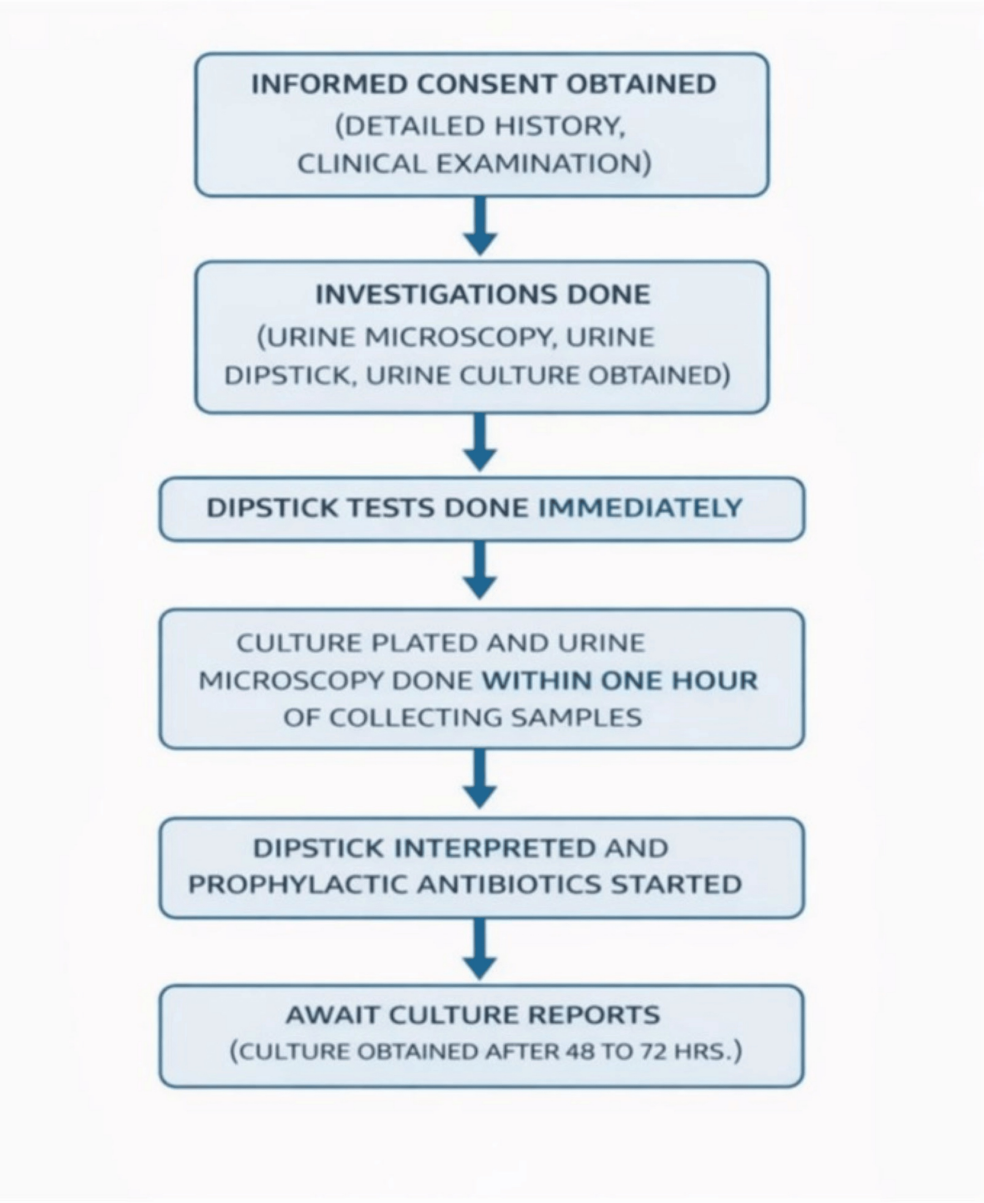
Flow diagram of patient enrolment and testing pathway

Children under two months, prior antibiotic use within 48 hours, or known immunodeficiency were excluded from the study. Female children presenting with vaginal discharge and menstruating adolescents were also excluded, as vaginal secretions or menstrual blood can cause transient haematuria, with even minimal blood producing macrohematuria and necessitating repeat sampling, which may delay treatment. The data collection procedure is shown in Figure 1.

After obtaining informed consent from the parents, a detailed history and clinical examination were conducted. It was followed by relevant investigations, including blood tests, urine dipstick, urine microscopy, and urine culture. Imaging was performed for children with positive urine culture results, and further investigations were carried out as per the IAP revised guidelines on the management of urinary tract infections, 2010.

The urine samples were collected in two 10-ml containers under strict aseptic precautions prior to antibiotic initiation for urine analysis and culture. For children under three years, catheterization was performed, while midstream clean catch was employed for those over three years. Contamination by periurethral or preputial organisms was minimized by washing the genitalia with soap and water and ensuring labia separation in female children. 

Urine dipstick test was performed immediately after sample collection using multistix strips to detect 10 parameters: nitrite, leukocyte esterase, pH, specific gravity, protein, glucose, ketones, urobilinogen, bilirubin, and blood. The strip was immersed in the sample, and colour changes were observed after 60 seconds for nitrite and 120 seconds for leukocyte esterase. 

Nitrite results were read as negative if there was no colour change and positive if a uniform pink colour was observed. Leukocyte esterase results were considered negative if there was no colour change and positive when any shade of purple was detected, graded from >1+. Colour changes were compared to the multistix bottle chart for interpretation.

Nitrite Test

This test detects nitrite in urine, produced by urinary gram-negative bacteria converting nitrate to nitrite with nitrate reductase. A minimum of 4 hours of urinary stasis in the bladder is required. Early morning freshly voided samples with nitrite positivity are highly specific for UTIs, with low sensitivity (50%) but high specificity (98%). The positive predictive value (PPV) was 66%, while the negative predictive value (NPV) was 18.5%. A positive test likely indicates a UTI with a significant increase in bacteria, typically exceeding 100,000/ml [8,14,15].

Leukocyte Esterase Test

This test detects leukocyte esterase (LE), an enzyme present in most white blood cells, except lymphocytes. It is released by neutrophils at the infection sites, and leukocyturia can be detected on a dipstick due to its esterolytic properties. It detects both intact and lysed neutrophils, making it more sensitive than pyuria alone. The test uses a calorimetric strip to measure esterase activity in the sample, providing a qualitative estimate of leucocyte count. The more neutrophils, the higher the esterase activity and the more intense colour change. The LE test can be positive even when microscopy shows no leukocytes, with leukocyturia being 84% sensitivity, 78% specificity with a PPV of 50% and NPV of 69.44% [15,16].

According to Urological Clinics of North America, if both nitrite and leukocyte esterase are positive on dipstick analysis, the sensitivity for detecting UTI is 80-90%, and the specificity is 60-98% with a PPV of 84-.6%. When both are negative, the negative predictive value is 100% [2].

Laboratory analysis

The urine specimen was promptly transported to the laboratory without any delay and plated on culture medium within an hour of collection to reduce false negatives. Microscopy was performed on centrifuged samples, and if processing was delayed, specimens were refrigerated at 4ºC for 12-24 hours. The culture identified causative organisms, distinguishing contamination, infection, and colonization as per the IAP protocol. Significant bacteriuria was defined in accordance with the 2022 guidelines of the Indian Academy of Pediatrics, as the presence of one or more bacteria per oil immersion field in a freshly voided, uncentrifuged urine sample, or by standard culture thresholds (≥10⁵ CFU/mL for clean‑catch specimens and ≥10⁴ CFU/mL for catheterized samples).

Samples showing contamination, such as the presence of blood or fecal material, with growth of more than one colony type, colony counts exceeding 100,000 CFU/mL, or acidic urine pH in the presence of E. coli were excluded from analysis. Equivocal cultures, defined as mixed bacterial growth or colony counts below the diagnostic threshold, were also excluded.

Patient factors such as hydration status, diet, prior antibiotic therapy, hematuria, and menstrual blood were considered before dip stic test. During specimen collection, samples were stored at appropriate temperatures and transported promptly to the laboratory. Dipstick‑related factors were also controlled by checking expiry dates, ensuring proper storage, and preventing contamination of reagent pads during testing.

The data obtained was entered in Excel and analyzed using R software version 3.6.1. All the categorical variables were expressed as frequencies and percentages. Laboratory parameters were evaluated for their diagnostic performance, including sensitivity, specificity, positive predictive value (PPV), and negative predictive value (NPV) in the diagnosis of UTI. Urine culture served as the gold standard for UTI diagnosis. The sensitivity, specificity, PPV, and NPV were calculated using standard formulas based on the values of true positives, true negatives, false positives, and false negatives. The Chi-square test was applied to assess the diagnostic accuracy of dipstick test results with urine culture findings. A p-value of less than 0.05 was considered statistically significant.

## Results

During the 12-month study period, a total of 140 children, suspected of having UTI as per the case definition, were enrolled in our study. Of which, about 53 (37.9%) were infants, 26 (18.6%) were aged between 13 months to three years, 27 (19.2%) were between 3 to 5 years, and 34 (24.3%) belonged to the above five years to 12 years age group. By gender, 68 (48.6%) were male children and 72 (51.4%) were female children.

**Figure 2:**
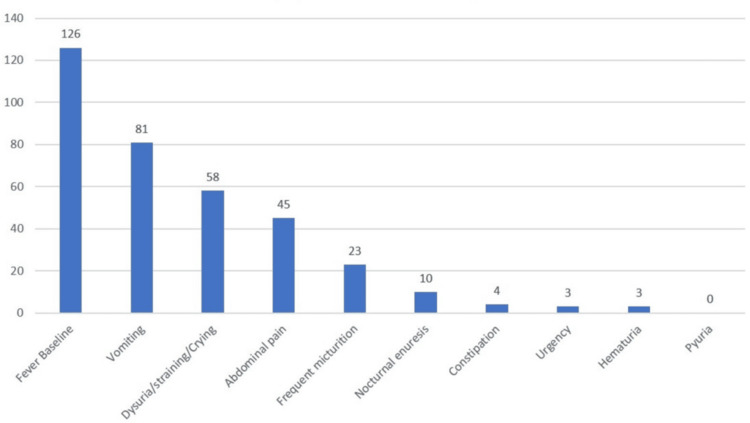
Clinical presentation of UTI in children aged 2 months to 12 years UTI: Urinary tract infection

The most common presentation was fever, which was present in 126 patients, comprising 90% of the study population, followed by vomiting (57.8%) and dysuria (41.4%). On examination, 97 (69.2%) children were febrile at the time of presentation, and 15 (10.7%) were found to have tachycardia (Figure 2).

Out of the total 140 patients, the urine culture was positive in 20 patients (14.3%) and negative in 120 patients (85.7%). Out of 20 children positive for UTI in urine culture, 16 were females, and four were male children. E. coli was the most common bacterium found in urine culture for 15 (75%) patients, followed by Acinetobacter, Klebsiella, Proteus mirabilis, and Staphylococcus epidermidis by 5% each. Of 20 children with significant bacteriuria, two children had USG abnormalities such as mild pelvicalyceal dilatation and mild hydronephrosis. All the new tests have been compared with the gold standard urine culture test for detecting UTI.

In Table 1, among the total 140 cases, nitrite was negative in 131(93.6%) patients and positive in nine (6.4%) cases. Of the 20 culture-positive cases, the nitrite test was positive in 6 cases and negative in 14 cases. The sensitivity, specificity, positive predictive value, and negative predictive value of nitrite were 30%, 97.5%, 66.7 % and 89.3%, respectively.

**Table 1:** Relationship between nitrite dipstick and urine culture in detecting UTI (N=140). The p-value obtained by the chi-square test is 0.000003, which is statistically significant. Values are presented as n (%), with percentages calculated based on the total sample size (N = 140) as the denominator. UTI: Urinary tract infection

Nitrite	UTI Culture		Total	Chi-square test
	Present	Absent	n (%)	
Positive	6 (4.3)	3 (2.1)	9 (6.4)	Test value = 21.56; p=0.000003
Negative	14 (10.0)	117 (83.6)	131(93.6)	
Total	20 (14.3)	120 (85.7)	140 (100)	

Among the 140 cases, Leukocyte esterase was negative in 111 (79.3%) patients and positive in 29 (20.7%) patients. Out of 29 patients who had leukocyte esterase positive, 9 patients showed 1+ colour change, eight patients showed 2+ colour change, and in one patient, 3+ colour change was noted. The remaining 11 patients had a leucocyte trace in urine.

Of 20 patients who showed culture-positive, leucocyte esterase correctly identified 11 cases and was negative in nine patients. The sensitivity, specificity, positive predictive value, and negative predictive value of leucocyte esterase were 55%, 85%, 37.9% and 91.9%, respectively (Table 2).

**Table 2:** Leucocyte esterase dipstick positivity in culture-positive UTI samples (N=140). The p-value obtained by the chi-square is 0.0004376, which is statistically significant. Values are presented as n (%), with percentages calculated based on the total sample size (N = 140) as the denominator. UTI: Urinary tract infection

Leucocyte Esterase Test	UTI Culture		Total	Chi-square test
	Present	Absent	n (%)	
Positive	11 (7.8)	18 (12.9)	29 (20.7)	Test value = 16.71; p=0.0004376
Negative	9 (6.4)	102 (72.9)	111(79.3)	
Total	20 (14.2)	120 (85.8)	140 (100)	

Table 3 shows that among the 140 cases, the combined dipstick was positive in seven patients and negative in 133 patients (95.0%). Among the 20 culture-positive patients, the combined dipstick was positive in six patients and negative in 14 patients. Among the 120 culture-negative patients, the combined dipstick was positive in one patient and negative in 119 patients. The sensitivity, specificity, positive predictive value, and negative predictive value of the combined dipstick compared with urine culture were 30%, 99.2%, 85.7% and 89.5%, respectively.

**Table 3:** Combined leucocyte esterase and nitrite dipstick positivity in UTI (N=140). The p-value obtained by the chi-square test is less than 0.0000001 and statistically significant. Values are presented as n (%), with percentages calculated based on the total sample size (N = 140) as the denominator. UTI: Urinary tract infection

Nitrite And Leucocyte Esterase Test	UTI Culture		Total n (%)	Chi-square test
	Present	Absent		
Positive	6 (4.3)	1 (0.7)	7(5.0)	Test value = 30.71; p=0.00000001
Negative	14 (10.0)	119 (85.0)	133(95.0)	
Total	20 (14.3)	120 (85.7)	140 (100)	

The urine microscopy for pus cells showed that pyuria was observed in 51 (36.4%) patients, and in 89 patients (63.6%), no pyuria was observed. Among the culture-positive group of 20 children, urine microscopy showed pus cells in 13 children and was negative in seven children. The sensitivity, specificity, positive and negative predictive value of pyuria were 65%, 68.3%,25.5% and 92.1%, respectively (Table 4).

**Table 4:** Urine microscopy for pus cells in culture-positive UTI (N=140). The p-value obtained by the Chi-square test is 0.004132, which is statistically significant. Values are presented as n (%), with percentages calculated based on the total sample size (N = 140) as the denominator. UTI: Urinary tract infection

Urine Microscopy	UTI Culture		Total n (%)	Chi-square test
	Present	Absent		
>5	13 (9.3)	38 (27.1)	51(36.4)	Test value = 8.23; p=0.004
<5	7 (5.0)	82 (58.6)	89(63.6)	
TOTAL	20(14.3)	120(85.7)	140(100)	

Further, the sensitivity, specificity, positive predictive value, and negative predictive value of Leucocyte esterase compared with urine microscopy are 37.25%, 88.76%, 65.52% and 71.72%, respectively. The Positive Likelihood ratio of leucocyte esterase was 3.3, which indicates that a patient with a positive test is 3.3 times more likely to have pyuria compared to urine microscopy.

Table 5 shows that urine microscopy has moderate sensitivity and specificity in diagnosing UTI. Nitrite test, as well as Leucocyte esterase test, have good specificity and negative predictive value in ruling out UTI. When compared to urine culture, the combination of both the nitrite and leucocyte esterase test has good specificity and negative predictive value to rule out UTI.

**Table 5:** Diagnostic accuracy of tests for pyuria among culture-positive UTI samples. UTI: Urinary tract infection; PPV: Positive predictive value; NPV: Negative predictive value

Tests	Sensitivity % (95% CI)	Specificity % (95% CI)	PPV % (95% CI)	NPV % (95% CI)
Urine Microscopy	65 (40.8-84.6)	68.3 (65.3-82.4)	31.7 (22.8-42.3)	92.1(86.5-95.6)
Nitrite	30 (11.9-54.3)	97.5 (92.8-99.5)	66.7 (35.2-88.0)	89.3 (86.2-91.7)
Leucocyte Esterase	55 (31.5-76.9)	85 (77.3-90.9)	37.9 (25.5-52.2)	91.9 (73.2-86.9)
Combined Nitrite and Leucocyte Esterase Positivity	30 (11.9-54.3)	99.2 (95.4-99.9)	85.7 (43.2-97.9)	89.5 (86.4-91.9)

## Discussion

The present study was conducted in children aged two months to 12 years to assess the role of urine dipstick in diagnosing UTI. In our study, fever was the most common symptom among UTI patients, followed by vomiting, dysuria, abdominal pain, frequent urination, loose stools, nocturnal enuresis, constipation, poor feeding, and lethargy. Previous studies done by Laskar AA et al., Kumar et al., Nayak et al., and Mod HK et al. reported similar findings in children [8,14,17,18].

In the present study, the sensitivity of urine microscopy for detecting pus cells was 65%, which aligns with the findings of Nayak et al. and Mod HK et al., but is lower than reports by Gabriella J Williams et al. and Fernandes et al. [8,14,19,20]. The specificity of 68.3% was higher compared to Nayak et al. and Mod HK et al. [8,14]. Additionally, while the positive predictive value was lower than that observed in other studies, the negative predictive value was comparatively higher. While the negative predictive value of urine microscopy and combined dipstick is comparable in our study, microscopy requires a laboratory setup and trained personnel, delaying decisions compared to the rapid dipstick test [19].

In our study, nitrite sensitivity was 30% with a high specificity of 97.5%. This pattern of low sensitivity but strong specificity has been consistently reported in several Indian studies. Mambatta et al. in Tamil Nadu documented a low sensitivity of 23.3%, while most studies reported values between 47% to 50 % [14,19,20,21]. In contrast, Cyril JP et al., in Kerala and Laskar et al., reported much higher sensitivities of 69.7% and 79.7%, respectively [17,22]. The variability in nitrite sensitivity is largely attributable to false negatives, which may occur if urine is retained for less than 4 hours because of frequent bladder emptying, or in cases of diluted urine and infections caused by non-reducing bacteria. Despite this variability, most of the studies reported a specificity of around 90% for the nitrite dipstick test [17,22]. Similarly, our findings confirm that nitrite testing, despite its limited sensitivity, offers high specificity and a reliable negative predictive value, useful in diagnosing pediatric UTI when positive, but its low sensitivity implies a negative result cannot exclude infection.

According to AAP norms, the sensitivity of leucocyte esterase was 83% (67-94%), higher than in our study, while the specificity was 78% (64-92%), lower than our findings [23]. Leukocyte esterase shows wide variability in sensitivity (40-80%) and specificity (25-81%) across studies [14,17,20-22]. Our study’s sensitivity (55%) was consistent with many reports, while specificity (85%) was comparatively higher. Although the positive predictive value (PPV) was lower than in some studies, the negative predictive value (NPV) remained high, reinforcing LE’s utility as a screening tool to rule out UTI rather than confirm it.

For combined dipstick testing, our study demonstrated low sensitivity (30%), high specificity, a good negative predictive value, and relatively favorable positive predictive values. Our findings aligned with a meta-analysis report of combined testing with 45% (30-61) sensitivity and 98% (91-99) specificity, indicating it as a useful screening tool for UTI [19]. A study done by Dadzie et al reported much lower sensitivity (16%), but high specificity with combined testing [24]. While most studies reported a sensitivity of around 60% [25], our results diverged by showing markedly lower sensitivity but the highest specificity (99.2%), consistent with Fernandes et al., Babu HJ et al., and Dadzie et al. [21,24,25]. Conversely, studies by Nayak et al. and Mod HK et al. reported the opposite pattern of low specificity with high sensitivity [8,14]. The positive predictive value in our study aligned with Fernandes et al., while the negative predictive value was comparable to Ruchika et al. [21,26]. Such variation would have occurred due to differences in patient population, sample handling and dipstick brand characteristics.

Therefore, in suspected pediatric UTI, a negative combined dipstick test with a high negative predictive value can reliably exclude infection in most patients, reducing unnecessary cultures, antibiotic prescriptions, and hospital visits. This triage strategy is especially valuable in resource‑limited settings, consistent with Indian studies and the Indian Academy of Pediatrics 2022 guidelines [27], which recommend dipstick use for early identification while awaiting culture. Similarly, the World Health Organization's Integrated Management of Childhood Illness (IMCI) framework and the National Institute for Health and Care Excellence (NICE) guidelines support point‑of‑care urinalysis to guide timely decisions in primary care [28,29].

While dipstick testing provides rapid screening, Gram stain offers higher sensitivity and specificity, whereas automated urine analysis ensures higher accuracy in detecting pyuria and bacteriuria. However, these technologies require infrastructure and cost investment, limiting their use in primary care and community settings [30].

Strengths and limitations

The strength of our study lies in its real-world application in resource-limited outpatient settings. It is good at ruling out infection, but there is a risk of missing cases as the sensitivity is poor. Key limitations include a relatively small sample size of culture positives and a single-center study, which may affect the generalizability of the findings. Additionally, the study does not place significant emphasis on the Gram stain technique for identifying bacteria, potentially overlooking its diagnostic utility. Further, children less than two years old were not taken into account, as the nitrite test is less reliable in infants due to frequent voiding and lower urine pH [29]. Stratification by vesicoureteric reflux (VUR), renal anomalies, and recurrent UTIs was not performed because those patients were excluded due to ongoing antibiotic prophylaxis. In addition, confounding factors such as sub‑preputial contamination in uncircumcised boys and chronic bacteriuria in children with vesicoureteral reflux or renal malformations could not be fully controlled. Age‑related immune response differences, dipstick brand and lot variability may also have influenced dipstick performance, limiting generalizability across all pediatric age groups.

Recommendations

In children with suspected UTI and a negative combined dipstick, UTI is unlikely, and culture may be deferred unless clinical suspicion remains high.

## Conclusions

The nitrite and leucocyte esterase dipstick tests, either individually or in combination, demonstrated good specificity and negative predictive value compared with urine culture. Although not a gold-standard test, the urine dipstick test is useful for predicting UTI in children. Therefore, they can be used as a valuable screening tool to rule out UTI in children, particularly in small outpatient clinics, primary health centres, community settings, and resource-limited environments. However, its low sensitivity carries a risk of missed cases. A high index of clinical suspicion and careful correlation with presenting symptoms are essential for its application in the community.
